# Future of Smart Cardiovascular Implants

**DOI:** 10.3390/s18072008

**Published:** 2018-06-22

**Authors:** Anubhav Bussooa, Steven Neale, John R. Mercer

**Affiliations:** 1 School of Engineering James Watt South Building, University of Glasgow, Glasgow G12 8QQ, UK; a.bussooa.1@research.gla.ac.uk (A.B.); steven.neale@glasgow.ac.uk (S.N.); 2 BHF Glasgow Cardiovascular Research Centre Institute of Cardiovascular and Medical Sciences, University of Glasgow, Glasgow G12 8TA, UK

**Keywords:** cardiovascular, atherosclerosis, implantable, smart, telemetry, communication, flexible, sensor, reporting, lithography

## Abstract

Cardiovascular disease remains the leading cause of death in Western society. Recent technological advances have opened the opportunity of developing new and innovative smart stent devices that have advanced electrical properties that can improve diagnosis and even treatment of previously intractable conditions, such as central line access failure, atherosclerosis and reporting on vascular grafts for renal dialysis. Here we review the latest advances in the field of cardiovascular medical implants, providing a broad overview of the application of their use in the context of cardiovascular disease rather than an in-depth analysis of the current state of the art. We cover their powering, communication and the challenges faced in their fabrication. We focus specifically on those devices required to maintain vascular access such as ones used to treat arterial disease, a major source of heart attacks and strokes. We look forward to advances in these technologies in the future and their implementation to improve the human condition.

## 1. Introduction

Implantable Medical Devices (IMDs) are in common use in medicine for diagnostic and therapeutic purposes [1]. They cover a diverse set of pathologies including glucose monitoring for diabetes, high blood pressure telemetry devices, cardiac reporters and defibrillators. Implantable medical devices are diverse and can interact with physiological processes such as the heart beat and mediate sensing and local stimulation, data recording and even drug delivery [2]. The latest IMDs can transmit important measurements such as blood pressure, blood glucose level and electrocardiogram from inside the body to the external world [3] many of these outputs can be used to aid clinical decision making and offer a personalised medical approach to diagnosis and therapy, yet their implementation faces significant challenges. Of particular interest is the potential of self-reporting cardiovascular stents. These bare metal cages are used to reopen blocked vessels caused by “furring-up” of the arteries. While this chronic disease is often fatal, patients “lucky” enough to be admitted to hospital with chest pain will often undergo angiography in which radiopaque dye is delivered to the fine coronary arteries of heart to detect narrowing’s that restrict blood flow and oxygen to the heart muscle—the myocardium. While cardiac by-pass is an option where left internal mammary arteries or saphenous veins taken from the legs are used as replacement conduit vessels, this is not a suitable option for all patients. The alternative is balloon catheterisation and delivery of the stent via the femoral arteries of the leg in which the device is threaded up to be deployed in the heart. Over 1 million implantable medical cardiovascular stents are implanted per annum in the USA and another million in Europe. The global market for medical devices is enormous and estimated conservatively at 8 Billion Euros in 2017, growing at 10% per annum.

Size, power and functionality constraints determine the design of an IMD and have limited their use for vascular stents thus far [4]. Developments in biotechnology, microelectronics and material science has now started to make it possible to overcome many of these constraints [5]. In recent years there have been significant advances in low power miniaturization of devices with highly integrated circuits (I.C.). Advances in microelectronics energy efficiency, ensuring long-life and improvements in biocompatibility and communication protocols have allowed systems with real-time vital monitoring to be implanted and to communicate wirelessly to report on chronic and acute medical conditions. In this review we provide key examples of the types of devices currently used in clinical practice and those more experimental that may eventually make it to market. While not possible to provide a comprehensive in-depth analysis of all devices, we offer an overview of the types available that are making a significant contribution to the clinical field. We explore the challenges in their methods of fabrication and the integration of sensors onto these often-intricate designs.

We focus explore implantable medicals devices and their method of powering including passive and wireless examples, compared to traditional charging and human energy harvesting systems. Communicating with IMDs and the complexities of microfabrication are important for the future success of these units. This area is investigated with examples of some of the latest techniques including printable and stretchable electronics in the context of cardiovascular disease, such as implantable blood pressure monitors in endovascular repair and coronary artery stents and leadless cardiac resynchronization therapy.

## 2. Implantable Medical Devices

IMDs can be classified as either passive or active. A passive IMD is a medical device that is not powered at all such as bare metal coronary artery stent [6]. An active IMD is “a medical device that is equipped for its functioning with a source of electrical energy and is totally or partially introduced, surgically or medically into the human body” [7]. The purpose of active IMDs is to monitor physiological or pathological signals and/or to induce therapeutic effects by delivering electric impulses to organs or tissues. In the case of coronary artery stents intimal hyperplasia (I.H.) often termed restenosis or thickening of the inside of the vessel frequently occurs as a consequence of delivery. This can occur in up to 15% of procedures despite the use of drug eluting stents that delivery chemotherapeutic drugs to inactivate cell growth. Integrating active IMDs to either transmit the signals they are monitoring to the external world and/or use it as input data for therapy is the “Holy Grail” of cardiovascular stent research. The application of each type is dependent on the application proposed. For example, cardiovascular stent technology would benefit from a passive device that doesn’t interfere with the intrinsic electrical properties of the heart, whereas more power intensive applications such as the blood pressure telemetry would benefit from a continuous high energy power supply.

## 3. Powering Implantable Medical Devices

While current cardiovascular stents are passive, requiring no electrical power, recent innovations in smart devices provide examples of how power sources can be integrated to achieve interrogating and transmitting effects to provide useful biological information. Smart devices often require a battery, which in many instances occupies a significant portion of the space required by the device [1,2]. One of the main advantages of battery powering is that it can provide a reliable continuous source of electrical current [8].

For example the first implantable cardiac pacemaker in the late 1950s utilised zinc/mercury oxide batteries [9] but are limited by their potential toxicity with the human body. Lithium/iodine became the standard for implantable pulse generators by the mid-1980s. However, in recent years cardiac pacemakers have become more sophisticated. They incorporate features such as memory to store electrical activity of heart and telemetry. The power requirements have led to implementation of lithium ion batteries with hybrid cathodes, which have higher power density. However, irrespective of the innovations in battery technology, the lifetimes of batteries will always be limited and thus would require replacement by periodic surgical interventions [2]. Advances in microelectronics now allow implants in the order of millimetres to be fabricated, but miniaturising the battery remains a significant challenge. In the context of cardiovascular stents, wireless powering is a more realistic long-term solution.

## 4. Wireless Powering

The battery size constraints makes wireless powering a very appealing possibility [10]. Instead of batteries, electrical coils can be used to receive energy from another coil outside the body. Until recently transferring energy beyond superficial depths in tissue required a coil of at least 1 cm in diameter. If the implant is in the order of millimetres, then the coil was thought to become unpractical. Yet, recent work by Ada Poon’s group at Stanford has developed high performance wireless powering systems that could be integrated into minute medical devices such as stents. Using micrometer scale magnetic resonance coupled radiofrequency identification (RFID) they have developed a series of wireless biosensors that can be powered and relay signals from diverse orientations that may overcome this previous limitation [10].

Some IMDs can be equipped with a rechargeable battery and a receiver coil [8]. A transmitter coil outside the body generates a magnetic field which transfers energy through the skin to the receiver coil. The magnetic field induces an alternating current (A.C.) in the receiver coil, and this is converted to a direct current (D.C.) by a rectifier. This rectified voltage can be used to power the smart stent or recharge the battery. A more elegant solution is using the body’s own waste energy for generating the minimal power required for low power smart stent devices.

## 5. Human Energy Harvesting

Human energy harvesting is used to define systems that use the human body as primary energy generator [11]. These systems capture gravitational, chemical, mechanical, thermal or electromagnetic energy and convert it to electric energy [12]. The main issue is that the energy yield is quite low. However, with the advent of ultralow power circuits, energy harvesting has become an attractive solution to powering implants [13]. Tashiro et al. [14] developed a “variable-capacitance-type electrostatic (VCES) generator that harnesses ventricular motion”. Its aim was to power a pacemaker permanently without a battery. They used a variable capacitor which converts mechanical energy from linear motion into electrical energy. The ventricular wall is chosen as the site of implantation because it has a large motion which occurs continuously. Their system was capable of producing 36 µW for powering a pacemaker for more than 2 h in an animal experiment. Clearly more work is needed to achieve either greater power outputs for sustained, long-term indwelling devices or advances in reducing power consumption of miniaturized devices thereby matching the needs of energy supply with energy consumption.

## 6. Communicating with Implantable Medical Devices

When it comes to wireless active smart stents and IMDs, the trade-off is between power consumption for high performance and power consumption for high data transmission rate [15]. This is because the power which can be transmitted to the implant antenna is directly proportional to the size of the latter. Moreover, regulations only allow transmission in the low-megahertz (MHz) frequency range, thus leading to the low data transmission rates and the low power efficiency. Recent studies [10,16] have shown that the power efficiency is optimal in the low-gigahertz frequency range, when using these millimetre-sized antennas. Optimisation in energy transfer protocols and data processing such as the use of fuzzy logic, neural networks and Bayesian interference through machine learning will all help to minimise these artefacts.

For example an active smart stent would be made up of biosensors, a wireless module and a miniaturised computer [17]. When the stent is implanted, it is configured according to the condition of the patient. If at a later time point, the medical condition of the patient changes, the wireless module allows changes in configuration in order to optimise monitoring and/or treatment. The stent can communicate with a remote server via a receiver [18]. The communication between stent and receiver is achieved through Medical Implant Communication Service (MICS). The receiver then communicates, via a wired network, with the server, which saves long term data. The possibilities for the communication protocol between IMDs and/or wearable medical devices are as follows: WLAN (wireless local area network), WPAN (wireless personal area network), LR-WPAN (low rate wireless personal area network) and WBAN (wireless body area network) [18]. The first three are more suitable for the on-body communication, while the latter is meant to be appropriate for the in-body communication.

Hsu et al. [18] proposed a new scheme called E2R MedRadio employing the WBAN communication protocol. The main objective of this scheme is to minimise energy consumption of the IMD by increasing communication reliability. The IMD uses much of its internal power for retransmission because of poor communication reliability. The latter can be enhanced by using group communication. The patient has an electronic wrist-ring and/or patch which acts as wearable device(s). In terms of communication protocol, the device is referred as IMD node, the wearable devices as wearable nodes and the main server as header node. The wearable nodes relay information from the IMD node to the header node. Thus, the IMD uses its energy for signal delivery only while the wearable nodes process the signal and relay to main server. However, achieving these designs is reliant on innovative fabrication techniques.

## 7. Microfabrication Techniques, Sensor Design

Microfabrication techniques which were first developed to produce integrated circuits have been modified to create sensors for biomedical devices. Electronic and optoelectronic sensors range from research devices that can electrically interface with brain tissue through densely packed microfabricated needles [19] to therapeutic devices such as FDA(Food and Drug Administration) approved retinal implants [20]. MicroElectroMechanical Systems (MEMS), defined as a semiconductor type device where a mechanical part works together with an integrated circuit, are a further development of microfabrication that then allows the detection of physical forces. This allows the creation of the devices that include accelerometers, strain gauges, microphones, air mass flow sensors and pressure sensors [21].

MEMS has allowed the manufacturing of smart medical devices small enough to be implanted in the human body for health monitoring, sensing and therapy [22]. As MEMS technology progresses, there will be further decreases in the size of the implants, which will also reduce invasiveness of the procedures required for implantation. An example of where MEMS has enhanced an implant is the cardiac resynchronisation therapy (CRT) device. It employs sensors for heart sounds in order to optimise resynchronisation. When the sensors were miniaturised they could fit into the pacemaker main body. Further miniaturisation allowed the sensor to be integrated into the tip of the leads in direct contact with heart wall. Thus, the small size of the new sensor increased the reliability, as it was in direct contact with organ requiring therapy.

The design and fabrication method is restricted by the material of choice [23] and for stents these are often composed of chromium/platinum composites and memory nickel titanium alloys such as nitinol. The latter satisfies the criteria of biocompatibility, hermeticity and mechanical strength/durability. Successful in vitro experiments using biocompatible materials does not guarantee successful operation following implantation, as the material can degrade with time and can also lead to immune responses that drive inflammation and implant rejection. Materials used for fabricating IMDs can be categorised as hard or soft. Other examples of hard biocompatible materials are, cobalt-chromium, iridium and certain glasses, while examples of soft biocompatible materials are poly(methyl methacrylate) (PMMA), polydimethylsiloxane (PDMS), Parylene C and polyimide and even the dissolvable substrates such as Abbott Laboratories’ GT1 stent made from poly-l-lactic acid (Abbott^TM^).

Due to concerns about electrochemical corrosion and immune reactions, micro-machined sensors and circuitry are enclosed within biocompatible materials [23]. Some of those materials also ensure hermeticity and electrical insulation. Silicon remains the material of choice for micromachining applications because of its compatibility with integrated circuitry (IC) and complementary metal–oxide–semiconductor (CMOS). Glass is the material of choice in IMDs that incorporate wireless interrogation. This is because glass provides electrical insulation of microfabricated metallic coils or antennae.

**Figure 1:**
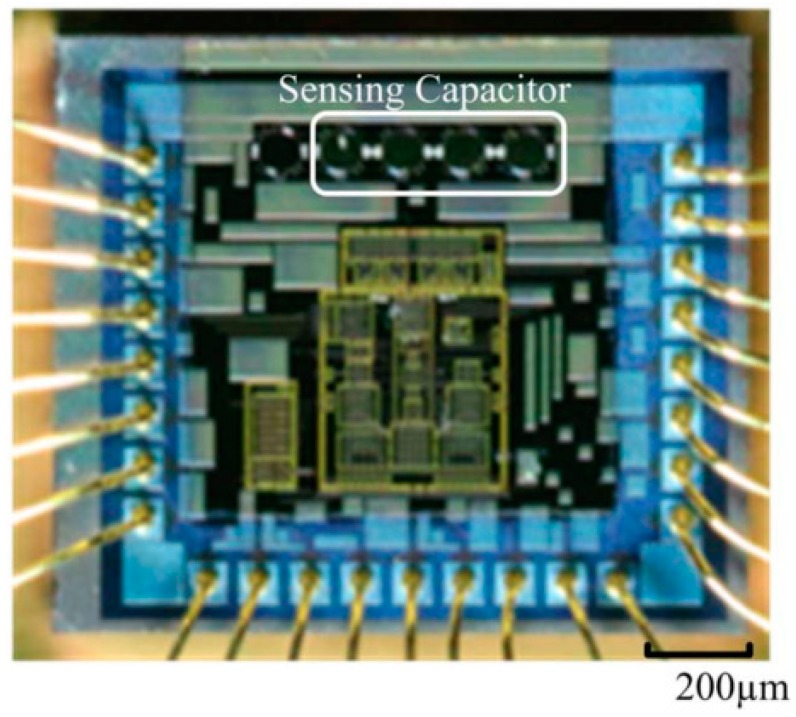
Micrograph showing sensing capacitor integrated on top a chip.

The sensor component(s) of a stent implant needs to be connected to an Application Specific Integrated Circuit (ASIC) in order to transmit the recorded signal to the external world [24]. The ASIC comprises a microcontroller, signal conditioning circuits and radio communication circuitry. Traditionally ASIC and sensors are assembled horizontally using Printed Circuit Boards (PCB). In order to reduce footprint of the sensing system, vertical stacking can be used (Figure 1). This type of convergent system is referred to as system-on-package (SOP) [25]. It incorporates system components at microscale using thin-film technologies. It benefits from the synergy between the integrated circuit, package and system. Tsai et al. [26] presented a micro-machined capacitive sensor with readout circuit on a single chip. However, integrating useful electronics sensors and therapeutic circuitry with medical devices is often limited by the traditional silicon materials used that have limited flexibility and are intrinsically brittle.

## 8. Printable and Stretchable Electronics

Stretchable interconnects, recently reviewed highlights advances made in overcoming the obstacle of silicon based materials [27]. Nanocomposite based inks for printing using either dispersed carbon or metal-based filler materials have become potential solutions. However, devices with large deformations such as smart cardiovascular stents require stretchable portions, sophisticated safe harbour locations for the non-flexible portions of the electronics or integrated silicon circuits to be protected. In essence, these devices will require the synthesis of engineered shapes and rubber-like stretchable materials such as polyurethane to achieve their goals of a personalised approach to medical devices. Integrating helical or serpentine wavy shapes at the milli and microscale will also help achieve this. Sekitani et al. [28] produced stretchable material using uniformly dispersed single walled carbon nanotubes (SWNTs). Innovative printing techniques also offers alternatives to soft lithography. The dispersed solutions of the conductors, dielectric and the semiconductor materials can be patterned onto the flexible substrates directly with a high resolution of only a few cells thick (314–286 μm) [29].

Materials which can balance mechanical stretch ability with electrical conductivity could overcome these issues of variable impedance for example. Polyaniline (PANi), poly(3,4-ethylenedioxythiophene): poly(4-styrenesulfonate)(PEDOT:PSS) and ionic hydrogels made of PAM-Aam (LiCl) all offer significant potential and have the advantage of being deformed up to 500% [30].

## 9. IMDs in Cardiovascular Disease

Cardiovascular disease (CVD) is a major cause of death globally [31]. In 2013, CVD contributed to 30% of global deaths or 17.5 million people. However, declines in population-based mortality rates have been observed in many countries since 1970 [31]. This decline can be attributed to better prevention of acute myocardial infarction and improved acute medical treatment. Thus, there is an increasing number of patients living with chronic CVD [32]. Over the last 20 years in United Kingdom (UK) surgical operations and medical treatment of CVD have significantly increased [33]. In 2012/2013, the National Health Service (NHS) spent approximately £6.8 billion on CVD, mostly on secondary care.

The ageing UK population exerts the significant financial pressures on the current healthcare system [34]. There is a need to reduce healthcare costs by minimizing inpatient hospital stays. The implementation of more IMDs with communication capabilities into healthcare practice could potentially relieve the strain on the health system. While smart cardiovascular stents could have the most significant impact there are precedents for IMDs that can transmit data to the external world, allowing caregivers to remotely access information about the physiological conditions of their patients and decide whether further medical or surgical intervention is required. Some of these have already reached regulatory approval [34] and have started a shift in the healthcare system from managing illness in hospitals to managing wellness outside through the very early detection and treatment of diseases prior to appearance of symptoms.

Cardiovascular IMDs can be regarded as the most sophisticated IMDs to date, for example defibrillators and pacemakers, are life-sustaining devices [35]. As these devices are implanted inside the body, it gives some level of autonomy to the patients, as they do not need to be hospitalised for long periods of time and can even be remotely monitored. Other devices such as implantable pressure monitors or implantable blood glucose monitors allow early diagnosis of low/high blood pressure or blood glucose respectively, allowing patients/caregivers to take action prior to appearance of symptoms. Here we provide examples of how far the technology has come.

### 9.1. Implantable Blood Pressure Monitors in Endovascular Repair

Some of the first implantable cardiovascular devices were used to treat abdominal aortic aneurysm (AAA). This is an age-related ballooning of the aorta, the main arterial vessel of the body. The vessel thins and becomes liable to rupture with often fatal consequences. The use of Endovascular Repair (EVAR) for treating AAA repair was first approved by the FDA in 1999 [36]. It is a stent-graft synthetic vascular mesh replacement and is used to exclude the aneurysm sac from systemic pressure. However, the major complication of EVAR is endoleak, which is recurrent high pressure within the aneurysm sac. It is very difficult to diagnose endoleaks using conventional imaging techniques, and thus measurement of sac pressure is carried out using an angiographic catheter [37]. However, this catheter cannot be kept inside the body for the long periods of time, and thus does not allow the long-term surveillance.

**Figure 2:**
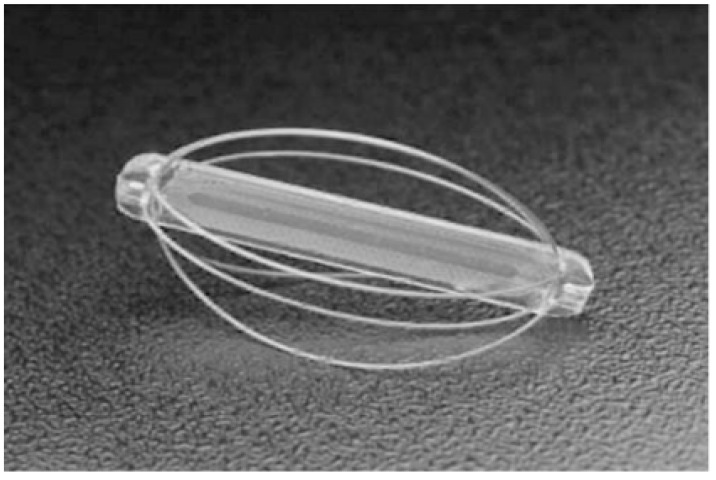
EndoSure pressure sensor (CardioMems).

The EndoSure sensor (CardioMEMS, Abbott Laboratories, Atlanta, GA. USA) (Figure 2) is a battery-less pressure sensing implant, which is deployed within the aneurysm sac and it is kept in place using the wire basket that surrounds it [37]. It is a passive IMD since it does not include any electronic components. It is simply an LC tank (inductive-capacitive tank). The inductive component is fixed, while the capacitive component consists of flexible plates whose separation varies with blood pressure [36]. As a result of the change in capacitance the resonant frequency (while transmitting radiofrequency) changes. The shifts in resonant frequency is transduced into real-time pressure measurements.

**Figure 3:**
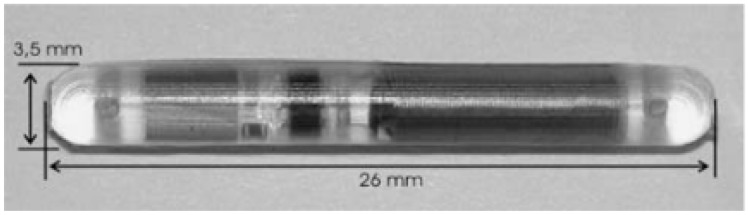
An abdominal aortic aneurysm (AAA) pressure sensor (Helmholtz Institute for Biomedical Engineering & Institute of Materials in Electrical Engineering, RWTH Aachen).

The Pulmonary Artery Pressure by Implantable device Responding to Ultrasonic Signal (PAPIRUS II) study [38] tested the feasibility of using the ImPressure device (Boston Scientific, Marlborough, MA, USA) for home monitoring of chronic heart failure (CHF). The device consists of an implant and an external unit. The implant comprises a pressure sensor, a piezoelectric transducer, a control chip and a battery (Figure 3). Ultrasonic energy is used to transmit a signal to the implant, activating its battery. The sensor measures full pressure waveforms for 10 s and the chip transmits the data to the external unit. The ImPressure device is fixed to the outside of the endograft by hand-sewing [36].

With both ImPressure and EndoSure, the signal transmission is analogue [39]. The data transferred can be influenced by surrounding signals and thus it is difficult to validate it. The Telemetric Pressure Sensor (TPS) (RWTH Aachen, Aachen, Germany) avoids surrounding interference by using digital signal transmission instead of analogue [36]. The TPS is in the form of a capsule which contains the required passive components (capacitors, receiver coil and zener diodes) and also a digital data processing unit. The repurposing of these technologies when applied to coronary stents would be transformative for cardiovascular disease.

### 9.2. Coronary Stents

The main purpose of coronary stents is to improve blood and oxygen perfusion to the heart and prevent arterial recoil following balloon angioplasty, by providing mechanical support to coronary arteries [40,41]. However, bare metal stents introduced in the mid-1980s frequently denuded the endothelial layer of the vessel during deployment and initiated an inflammatory reaction that promoted vascular smooth muscle cell proliferation and clot formation that could re-block the vessel. Advances in drug eluting stents with polymer timed release coatings of cytotoxins such as sirolimus and paclitaxel were developed and FDA approved but the problems persist. For example, antiplatelet medications such as clopidogrel reduced the blood clot risks, but left patients susceptible to bleeds. In addition the latest stents have led to a different complication such as malapposition where the stent fails to contour to the vessel wall can promote thrombosis and in-stent restenosis (ISR) [42]. In current practice, repeat angiography is the only method used to diagnose ISR, but the accuracy of the clinical assessment is low [43]. Clinically it is impossible to know what the stent is doing inside the vessel hence alternative diagnostic methods are required to stratify the patient populations and augment therapies.

Several studies [43,44,45,46,47,48,49,50,51,52,53] have all attempted to find reliable ways of diagnosing ISR by integrating additional components onto the stent. Due to restenosis, there is a local increase in pressure within the stent and this can be detected by a pressure sensor. Most commercially available stents have a mesh-like tube design. Brox et al. [52] redesigned a stent into the form of a continuous helical coil and integrated a capacitive blood pressure sensor, so that the whole structure could function as an LC (inductive-capacitive) tank, allowing wireless interrogation. This stent-pressure-sensor system thus works as a passive IMD. The stent (inductor) and sensor (capacitor) combination is inductively coupled to an external coil, and the phase-dip is measured. Schachtele [44] modified this approach by integrating two pressure sensitive transponders at either ends of the stent. The delay between resonances of the two transponders is used to calculate the local pulse wave velocity. Son et al. [50] developed a stent with electronic components, which thus functioned as an active IMD. In addition to providing mechanical support, the smart stent had diagnostic and therapeutic capabilities. The stent and all the added components were fully bioresorbable. The stent integrated a blood flow sensor, a temperature sensor and an antiproliferative drug stored in a nanoparticle shell. The release of the drug was controlled photothermally.

Integrating electronic components onto a stent is a major challenge because these components need to resist stent crimping procedures and stent expansion [54]. Moreover, since the stent remains inside the patient’s artery for the rest of his/her life, the stent with electronic components needs to resist chemical and biological reactions. Chen et al. [54] developed a smart stent compatible with routine crimping and deployment procedures. A pressure sensor was securely integrated onto the stent using laser-microwelding. To ensure biocompatibility and X-ray opacity, Parylene C and gold coatings were applied. The device was demonstrated to successfully monitor in-stent blood pressure in a swine model.

### 9.3. Leadless Cardiac Resynchronisation Therapy

Conventional cardiac resynchronisation therapy involves the insertion of a transvenous lead, which runs from the pacemaker to the pacing site [55]. The major complication with this approach is lead failure, which occurs in 21% of patients within 10 years after implantation [56]. Other complications include the infection, tricuspid valve insufficiency and central vein obstruction [57]. The possibility of implementing leadless pacing has been considered for many years and has been shown to be feasible [58].

Leadless pacing can be achieved in two main ways [57]: (i) multicomponent devices, which consist of a subcutaneous energy transmitter and an endocardial pacing electrode; (ii) self-contained devices, which consist of a pacing electrode and pulse generator.

In the multicomponent design, energy needs to be transferred wirelessly from the transmitter to the receiver unit. This can be achieved via ultrasound-mediated energy transfer or electromagnetic induction. The Wireless Chronic Stimulation-Left Ventricle (WiCS-LV) system utilizes the acoustic energy [59]. It is co-implanted with a CRT device, ICD (implantable cardioverter defibrillator) device or pacemaker, and allows bi-ventricular pacing.

WiCS-LV detects the right ventricular pacing signal from the co-implanted device. The pulse generator then generates ultrasonic energy. This is received by the receiver unit (implanted in left ventricle), which converts the ultrasonic energy into an electrical pulse. Thus, the system allows synchronous right and left ventricular pacing. The energy transfer to the receiver unit is optimised by using beam-forming.

Wieneke et al. have shown that the energy transfer can also be achieved using electromagnetic induction [58]. The subcutaneous transmitter unit consists of a coil which sends an alternating magnetic field. This transmitter unit induces a voltage in the receiver unit which consists of a coil with a ferrite core, a bridge rectifier and a high impedance lead tip. Electromagnetic resonance coupling allows optimisation of the energy transfer. However, the energy transfer efficiency is affected by movement of the receiver unit due to beating of the heart.

## 10. Discussion

The global population is continuing to live longer with more complicated medical conditions with multiple co-morbidities. The associated care costs and increasing susceptibility to chronic diseases will ensure the need for new and innovative devices with advanced medical diagnostic and therapeutic properties. Interdisciplinary research continues to push the boundaries of what is possible and develops high tech devices which integrate advanced sensor design, fabrication and manufacturing techniques. Those devices that make it to market will transform healthcare as we know it and will have broad appeal across healthcare disciplines from cardiovascular to neurological and renal pathologies. They will offer a personalised and stratified approach to medicine and will shift the healthcare paradigm from purely diagnostic to therapeutic. With the collection of large datasets to validate their use there is the opportunity for these devices to start a revolution in preventive medicine.
